# 

**Published:** 1891-07-18

**Authors:** 


					in tun " i,u.nun 11. xaau, baaui* LU.BHI ukp ^ .mBFiWSg^rsi
. price with a preparat. > combining in itself the albuminous together with tha extractive principles, such a preparation
* would have to be preferred to the Extractum Oarnis, for it would contain all the nutritive constituents of meat." Again?" I have
v>nfnrn stated that in pre- \W> paring the Extract of Meat, the Albuminous principles remain
in the residue, they are Wf lWl lost to nutrition, and this is certainly a great disadvantage.*'
mm mm  ?? BOV&IL " contains ^\M\ HI /M^ t be albumen and fibnne in the most perfect possible form, and to
?TOa^SSSBKE?"^ m SOLD THROUGHOUT THE UNITED KINGDOM^
it
251. Vol. x.l Saturday,
July 18th, 1891.
[One Penny.

				

